# Localized accumulation of kappa restricted Russell body‐containing plasma cells in tonsil

**DOI:** 10.1002/ccr3.2372

**Published:** 2019-08-09

**Authors:** Maryna A. Vazmitsel, Katsiaryna Laziuk, Richard D. Hammer

**Affiliations:** ^1^ Department of Pathology and Anatomical Sciences University of Missouri School of Medicine Columbia Missouri

**Keywords:** clonality, light chain restriction, plasma cells, Russell body, tonsil

## Abstract

An abnormal clonal plasma cell proliferation with Russell bodies is rare in chronic inflammatory reactions in adult patients. We describe the first case of light chain restricted Russell body accumulation within germinal centers of lymphoid follicles of the tonsil in a child. This should not be confused with a neoplastic process.

## 1 INTRODUCTION

Russell bodies were first described in 1890 as 4‐ to 12‐micron fuchsinophilic structures in areas of round cell infiltration of a variety of neoplasms and suggested to have a role in cancer.^1^ It is now known that Russell bodies are an abnormal collection of immunoglobulins within dilated rough endoplasmic reticulum cisternae of plasma cells that have accumulated secondary to abnormal secretion.^2^ Besides accompanying B‐cell lymphomas with significant plasma cell proliferation, plasma cells containing Russell bodies can be seen in chronic inflammatory infectious reactions with prolonged antigenic stimulation such as Klebsiella rhinoscleromatis‐induced Rhinoscleroma^3^ or Helicobacter pylori‐induced gastritis.^4^

Sometimes accumulation of plasma cells with Russell bodies may simulate neoplasm by itself. The first mention of an abundant accumulation of Russell bodies mimicking tumor was reported by Tazawa and Tsutsumi in 1998, when they described such a tumor‐like lesion of the gastric mucosa.^5^ In addition to the gastric mucosa,^6^, ^7^, ^8^ localized tumor‐like collections of the Russell bodies can be seen in the duodenum,^9^ esophagus,^10^ cervix,^11^ and skin.^12^ Russell body aggregates were reported to occur in 80% of the chronic periapical lesions in association with dental pulp^13^ as well as in “plasma cell granulomas” of sinuses and gingiva.^14^ The vast majority of cases of Russell body accumulation were reported in adult with only few pediatric cases.^15^

Of note, Russell body accumulation has never been reported in the tonsils in spite of active immune function of the organ.

## 2 CASE REPORT

A 2‐year‐old boy with a 6‐months history of severe obstructive apnea underwent an elective bilateral tonsillectomy. There were no other significant medical problems, and his immunizations were current with standard schedules.

The tonsils were surgically removed and were equal in size and shape with a tan‐pink homogenous cut surfaces and no mass lesions.

Formalin‐fixed paraffin‐embedded tissue was examined on 2‐microns sections stained with H&E and PAS. Immunoperoxidase studies with appropriate controls are performed with the following antibodies: CD3, CD4, CD8, CD19, CD20, PAX5, MUM1, CD30, CD138, kappa, lambda, IgG, IgM, and IgA.

**Figure 1:**
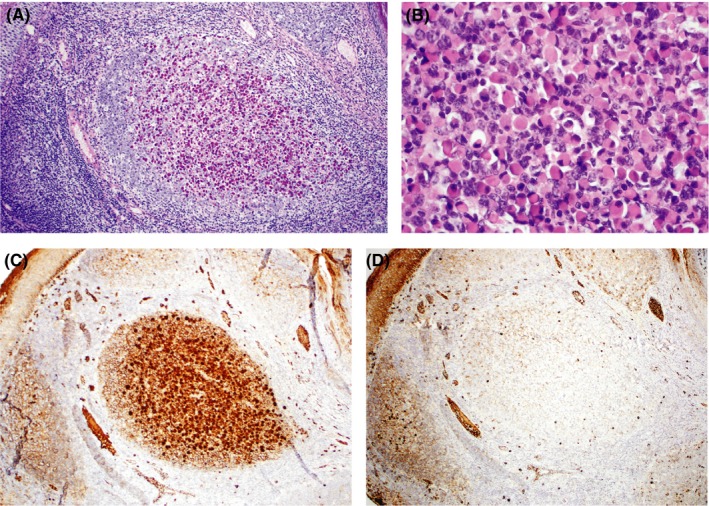
Germinal center accumulation of Russell bodies within the tonsil. A, Hyperplastic follicle with a nodular focus of plasma cells containing Russell body (PAS stain, original magnification ×200), B, High‐power view of plasma cells with prominent round intracytoplasmic inclusions (H&E stain, original magnification ×600); C, Kappa immunostains show a clonal population, and D, Lambda stains are negative (×100)

Sections of the tonsil reveals numerous hyperplastic lymphoid follicles varying in size and shape with germinal centers containing tingible body macrophages and well‐defined mantle zones. The interfollicular area consisted of small‐ and intermediate‐sized lymphocytes, plasma cells, histiocytes, and occasional immunoblasts. Crypts were preserved without ulceration of the tonsillar mucosa. Further study of the sampled tonsil disclosed collections of plasma cells within germinal centers of two follicles with one showing significant enlargement. At higher magnification, plasma cells in aggregates were characterized by eccentrically located nuclei and abundant bright eosinophilic cytoplasm that is stained bright‐pink with PAS—features consistent with Russell bodies (Figure 1A,B). The Russell body‐containing plasma cells expressed CD19, CD138, and MUM1 and showed monotypic IgM kappa restriction (Figure 1C,D). Plasma cells were negative for CD3, CD4, CD20, PAX5, CD30, and did not express lambda, IgG, and IgA. Serum immunoglobulin levels were not performed or available for review.

## 3 CONCLUSION

To our knowledge, this represents the first described case of Russell body accumulation within the germinal centers of lymphoid follicles of the tonsil of a child and the first reported example of Russell body accumulation with kappa light chain restriction in the tonsil.

The presence of monoclonal plasma cells with Russel body accumulation raises consideration of a plasma cell neoplasm but was excluded due to lack of evidence of plasmacytosis in bone marrow sites or clinical features of plasma cell myeloma, including renal insufficiency, anemia, or hypercalcemia. Light chain restriction is not common in benign or reactive processes, but has been occasionally reported with no associated overt gammopathy or hematopoietic malignancy, most frequently in the GI tract of adults.^9^, ^16^ To date, the kappa or lambda restricted Russel body polyps, gastritis, or gastroenteritis are well documented but polyclonal are more common.^5^, ^16^, ^17^, ^18^, ^19^ Recently, kappa clonal restriction of Russell bodies was reported in two cases of pediatric gastroenteritis.^15^ Both patients had immune dysregulation evidenced by GVHD and celiac disease. Given the mutational events that precede mature immunoglobulin formation, it is possible that in predisposed individuals, abnormal overstimulation of immune cells increases the likelihood, albeit randomly, of abnormal immunoglobulin production.

In this case, the plasma cells with Russell body accumulation are limited to germinal centers only. We believe it could be explained by the blockage of immunoglobulin secretion accompanied by arrest of activated plasma cells moving outside the germinal center of follicles. Most of the previously reported cases were in settings predisposing to chronic antigenic stimulation. While we considered chronic antigenic stimulation from recent immunization might be a predisposing factor here, specific dates of immunization were not available. Accumulation of activated plasma cells with Russell bodies within the germinal center of lymphoid follicle could be a manifestation of an underlying immune dysregulation or chronic antigen stimulation even in the absence of known immune abnormality.

The real incidence of Russell body accumulation in the tonsils is unknown as often pediatric tonsils are only randomly sampled, but it should be kept in mind to prevent over diagnosing as a tumor. Presence of aggregates of plasma cells with Russell bodies demonstrating kappa light chain clonality should not be mistakenly interpreted as a tumor, especially with the absence of additional morphological and clinical features.

## CONFLICT OF INTEREST

None declared.

## AUTHOR CONTRIBUTION

MAV: responsible for pathology review, preparing illustrations, reading of the appropriate literature, manuscript composition and answering editorial comments. KL: responsible for study design, pathology review, patient management, reading of the appropriate literature, composition of the manuscript and, answering editorial comments. RDH: responsible for study design, mentoring the study, pathology review, preparing illustrations, patient management, reading of the literature, composition of the manuscript and, answering editorial comments.
